# Harnessing the power of artificial intelligence for disease-surveillance purposes

**DOI:** 10.1186/s12919-025-00320-w

**Published:** 2025-03-06

**Authors:** Barbara Tornimbene, Zoila Beatriz Leiva Rioja, John Brownstein, Adam Dunn, Sylvain Faye, Jude Kong, Nada Malou, Clara Nordon, Benjamin Rader, Oliver Morgan

**Affiliations:** 1 World Health Organization Hub for Pandemic and Epidemic Intelligence, Berlin, Germany; 2 CPC Analytics, Berlin, Germany; 3 Boston Children’S Hospital, Boston, MA USA; 4 University of Sydney, Sydney, Australia; 5 Global South AI for Pandemic & Epidemic Preparedness & Response Network (Ai4pep), Toronto, Canada; 6 Medecins Sans Frontières (MSF), Paris, France

**Keywords:** Artificial intelligence, Public health surveillance, Infectious disease, Data privacy, Multidisciplinary collaboration

## Abstract

The COVID-19 pandemic accelerated the development of AI-driven tools to improve public health surveillance and outbreak management. While AI programs have shown promise in disease surveillance, they also present issues such as data privacy, prejudice, and human-AI interactions. This sixth session of the of the WHO Pandemic and Epidemic Intelligence Innovation Forum examines the use of Artificial Intelligence (AI) in public health by collecting the experience of key global health organizations, such the Boston Children's Hospital, the Global South AI for Pandemic & Epidemic Preparedness & Response (AI4PEP) network, Medicines Sans Frontières (MSF), and the University of Sydney. AI's utility in clinical care, particularly in diagnostics, medication discovery, and data processing, has resulted in improvements that may also benefit public health surveillance. However, the use of AI in global health necessitates careful consideration of ethical issues, particularly those involving data use and algorithmic bias. As AI advances, particularly with large language models, public health officials must develop governance frameworks that stress openness, accountability, and fairness. These systems should address worldwide differences in data access and ensure that AI technologies are tailored to specific local needs. Ultimately, AI's ability to improve healthcare efficiency and equity is dependent on multidisciplinary collaboration, community involvement, and inclusive AI designs in ensuring equitable healthcare outcomes to fit the unique demands of global communities.

## Introduction

During the COVID-19 pandemic, some Artificial Intelligence (AI) initiatives were developed with the aim of tracking, managing, and containing the spread of the disease [1–3]. With varying levels of success, these initiatives have shed light on the potential of AI in detecting and responding to public health outbreaks [2, 3]. In this paper, AI refers to a range of methods and applications, including machine learning algorithms, natural language processing, predictive modelling, and image recognition. These technologies have been used for tasks such as disease surveillance, resource allocation, diagnostics, and data synthesis to support decision-making and public health interventions.

Nevertheless, the implementation of AI in public health surveillance comes with challenges [4, 5]. Data privacy and the operationalization of the human-AI interaction are critical considerations [6]. As the AI training data are predominantly from sources in the Global North, we need to understand how to deal with responses that could carry biases from this perspective [7]. Additionally, ensuring seamless collaboration between AI systems and human public health professionals will be crucial to fully leverage the strengths and capabilities of both [8].

In this session of the World Health Organization (WHO) Pandemic and Epidemic Intelligence Forum [9], co-hosted with Boston Children's Hospital, the focus was on examining specific use cases of AI-assisted technologies in public health. The objectives of the forum were to explore specific use cases of AI-assisted technologies in public health, identify challenges and opportunities for implementation, and foster cross-sectoral collaboration to address ethical and operational issues. We brought the experience of the Global South AI for Pandemic & Epidemic Preparedness & Response Network (AI4PEP) and its partner Cheikh Anta Diop University in Dakar, Médicines Sans Frontières (MSF), and the University of Sydney to explore how these technologies, along with others, can complement the efforts of global health surveillance workers during future pandemics.

## Early days: AI and clinical medicine

The world of healthcare is filled with time-consuming tasks, particularly when it comes to handling vast volumes of clinical information. Whether it is about billing processing or making critical clinical decisions, the challenges are significant. AI models can operate behind the scenes, assisting in the delivery of care and facilitating clinical decision-making across various domains, from radiology and pathology to the intensive care unit [8]. For instance, advances in voice and language processing are revolutionizing physician–patient conversations. These interactions now flow more naturally, with the dialogue captured, processed, and integrated into electronic medical records [10]. The development of generative AI and large language models will further accelerate progress in this arena.

In addition to operational and administrative applications, AI can harness clinical data to improve healthcare research and delivery. By analysing vast clinical datasets, AI algorithms can quickly identify potential new drug candidates [11]. Furthermore, automation is streamlining the processing of clinical data, enriching electronic medical records, and reducing the burden of mundane tasks in day-to-day healthcare operations. This significantly reduces the research and development timeline, potentially leading to faster access to life-saving medications. The field of predictive disease mapping and capacity planning offers tremendous potential, as it enables the ability to forecast patient admissions, diagnose conditions more accurately, and explore a multitude of opportunities for improvement in healthcare [10, 12].

Another potential of AI is the generation of synthetic datasets, which are based on real data but contain no actual patient data, minimising risks to data privacy while enabling easier sharing [13]. Having good synthetic datasets will help develop new analytic and data processing applications for infection control and epidemic intelligence. This approach could open up value that has limited availability in currently restricted data frameworks.

Applying strategies and technologies inspired by clinical health practices could potentially improve efficiency and effectiveness of public health systems [14]. This convergence of clinical insights and cutting-edge technologies not only enhances the capacity to address health challenges but also paves the way for a more inclusive and patient-centric approach to public health.

## AI applications in infectious disease surveillance

In the past two decades, the work of the team at Boston Children's Hospital has focused on applying natural language processing models to specific areas of clinical medicine. In 2023, the team conducted a landscape analysis of the current applications of AI in infectious disease surveillance [15]. One identified interesting example is the way the Greek government employed AI to enhance surveillance and effectively deploy resources for disease control during the COVID-19 pandemic. They used AI to categorize incoming travellers into three groups: low information, low risk, and high risk of infection. Given the limited testing capabilities, the AI system helped determine whom to prioritize for testing: those classified as high risk and low information. This approach created a feedback loop where the AI system could collect data from individuals with low information, administer PCR tests to understand their risk profile, and improve the predictive capabilities of the model for future assessments. This innovative use of AI showcases its potential to optimize resource allocation in disease control efforts.

Boston Children's Hospital itself has utilized AI to study how epidemics impact communities. For example, during the winter 2022–2023, when paediatric infections surged and disrupted hospital operations in the US, AI tools helped to better understand when infections were occurring. By combining expert forecasts with data on different viruses circulating in the country, the AI models generated accurate local and regional forecasts for the hospital. Clinicians and planners were provided with valuable insights a few days or even a week in advance of an impending wave of infections, such as Respiratory Syncytial Virus and influenza. AI tools can also help in this regard by effectively identifying and differentiating pathogens within noisy data sets.

By using AI as a catalyst, it is also possible to promote interconnectedness of human, animal, and environmental health, adhering to the principles of the One Health approach. For example, the Cheikh Anta Diop University is implementing a project titled "Artificial Intelligence and Hybrid Modeling for Community-Based Early Detection of Zoonotic Diseases in the Context of Climate Change in Senegal" [16]. This hybrid model integrated data from diverse teams, including socio-anthropological and epidemiological units, along with community-based research, with the aim of enabling early detection and prevention of zoonotic diseases exacerbated by climate change. The Integration of multiple data streams will ultimately improve predictive machine learning models for one health applications.

## The power of AI in data extraction

The transformative impact of recent AI advancements, particularly the availability of large language models and related tools, has prompted a re-evaluation of information mining practices from traditional and untraditional data sources [17].

For many years, the team at Boston Children's Hospital has been mining online open data sources and extracting insights from unstructured data to gain valuable insights and inform public health decisions. They have incorporated data from surveys and participatory surveillance, all while developing tools to extract valuable insights from government reports and leveraging social networking sites. However, the advent of large language models and related tools has prompted a re-evaluation of how they approach data exploration.

HealthMap, an initiative that over the past two decades has been dedicated to organizing global information on emerging infectious diseases, can be used as an illustrative example. Its core concept involved scanning for keywords in various languages across hundreds of thousands of sites and employing highly specific classification systems for information organization. Over the years, the focus was on creating classification systems that could identify various elements such as location, disease counts, and the diseases involved. The emergence of large language models like ChatGPT has revolutionized this landscape [18]. The near future will rewrite established practices because it will soon be possible to input an article into these tools and generate summaries with a level of detail and classification with a speed never achieved before. This transformation will not only enrich historical information but open vast new opportunities for data exploration and the extraction of additional attributes.

In this context, the University of Sydney, affiliated with the Boston Children's Hospital Computational Health Informatics Program, has been exploring applications of Natural Language Processing (NLP) within the field of information surveillance for nearly a decade, with a particular emphasis on identifying and understanding the dynamics of misinformation. In the realm of information surveillance, a significant challenge lies in accessing data from social media platforms. Research often centres on identifying relevant posts tagging keywords or hashtags from individual social media platforms, and access to these data has become increasingly restricted or costly. Even with access to these data, it is challenging to connect longitudinal data about information exposure with outcomes such as diagnoses, attitudes, and behaviours.

For this reason, the team in Sydney shifted their focus toward refining methods that incorporate the use of machine learning to gauge people's information consumption, including methods to identify risk signatures associated with certain attitudes or behaviours. Health-related information originates from diverse channels, including online sources, but also offline channels such as conversations with health professionals. Establishing connections between information exposure and health behaviours required an innovative approach. The team developed a research platform that includes an information diary tool that study participants use to detail their exposure to information related to a specific health topic, covering sources, search history, chance encounters, and trust levels. By obtaining informed consent and incorporating the diary in studies, researchers can then explore which exposures are most closely associated with certain attitudes and behaviours, using validated survey instruments.

## The use of AI to enhance public health surveillance

For the past decades the integration of AI in clinical medicine has been revolutionizing diagnostics [10]. The power of AI in diagnostics can become beneficial also for surveillance [12]. One example is the way MSF is leveraging AI to fight antimicrobial resistance in settings where human resources such as microbiologists are not available. They developed “Antibiogo”, a cost-free, offline-capable smartphone application to measure and interpret Antimicrobial Susceptibility Tests (AST), the test that determines the sensitivity of bacteria to different antibiotics [19]. For years the organization has been conducting laboratory assessments and establishing collaborations with public health laboratories to enhance AST quality. Their experience revealed training technicians to perform ASTs was straightforward, but interpretation of results posed challenges that were mainly due to the absence of qualified microbiologists. “Antibiogo”,classified as a software medical device, uses AI for antibiotic identification and image processing for semi-automatic inhibition zone measurement. While final interpretation still relyies on a technician’s judgment, the device provides assistance through an expert algorithm that incorporates all interpretation rules from EUCAST and CLSI [20, 21]. Results are then presented in a user-friendly format for clinicians and nurses to ensure effective Infection Prevention Control and to inform clinicians' patient treatment decisions. Additionally, this software allows for the collection of quality ensured data that countries can use for surveillance purposes, for example by plugging it into more complex laboratory information systems like WHONET, used to collect and analyse surveillance data at local, national and global level [22]. Countries can subsequentially compile these data at national level and use them to report to regional and global Antimicrobial Resistance surveillance systems (e.g., WHO-GLASS).

Looking ahead, it is becoming clear that AI's role in diagnostics will vary across countries, tailored to their specific needs. For instance, in some contexts, AI may supplement laboratory specialists by performing assisted diagnostics, enabling more agile diagnostic processes. This, in turn, can facilitate more inclusive data collection from rural or geographically dispersed populations, ultimately improving machine learning models and enhancing public health surveillance. Conversely, in well-equipped settings, the adoption of AI is often motivated by the need to optimize resources and expand diagnostic access. This is particularly relevant in centralized health systems, where nimble AI solutions may be preferred over costly diagnostic machinery.

## Can AI boost multidisciplinarity and equity in public health?

The advancement of AI can usher in a transformative era within the realm of global health [5]. Through the use of machine learning models trained to analyse complex datasets, AI can enhance predictive modelling, and decision support, to catalyse disease surveillance, insightful data exploration, precision diagnostics, and streamlined resource allocation. Additionally, AI can also enhance multidisciplinary approaches by processing diverse data, revealing patterns across disciplines, and aiding informed decisions. It can advance equity by identifying disparities, tailoring interventions, and optimizing prioritization and distribution of resources [12, 15].

In this context, the main objective of the Global South AI for Pandemic & Epidemic Preparedness & Response network [16] is to enhance the understanding of responsible AI solutions to improve public health preparedness and response to the emergence and re-emergence of infectious diseases. Bringing together 16 countries, the network focuses on bridging knowledge gaps, building capacities, and creating innovative solutions. They are committed to influencing national, regional, and global policies and practices, harnessing the potential of AI to improve health equity and fortify public health systems through the development of proactive tools. By prioritizing health and well-being for all, as well as gender equality, they collaborate closely with public health authorities to ensure the utilization of AI for timely and reliable data to address the specific needs of vulnerable groups and communities, thereby enabling inclusive and equitable healthcare systems. For example, by applying local knowledge to the AI algorithm for COVID-19 spread to create digital dashboards for policy makers and communities.

The Cheikh Anta Diop University model is also based on multidisciplinarity and equity. The project aims to establish a community-oriented, gender-sensitive methodology, leveraging the potential of AI to enhance disease surveillance. It underscores the importance of involving local communities and integrating their insights and data into the framework to develop accurate and comprehensive solutions. This strategy is built upon five foundational pillars: community engagement through partnerships and participatory research, adopting a one-health perspective that amalgamates ecological, societal, and disease-related factors, employing technological tools and epidemiological modelling for the community-focused system, conducting ethnographic investigations into gender dynamics, and evaluating locally customized and ethically sound algorithms for public health applications. The project also emphasizes policy advocacy to foster collaboration with stakeholders. Ultimately, the goal is to establish a sustainable, all-encompassing one-health surveillance mechanism empowered by AI, guided by a gender-sensitive approach.

## Future steps for the responsible use of AI

Clear frameworks should be put in place so that AI solutions follow fundamental values that include accountability, fairness, transparency, reliability, ethics, security, inclusivity, and sustainability, forming the foundation for effective solutions that cater to communities' needs and respect.

First and foremost, it is crucial to consider the ethical implications and balance the benefits of AI with safeguarding people’s rights. AI, can be used to enhance surveillance capabilities, while still prioritizing the protection of individuals' data. An illustrative example is Global.health[23], a collaborative data science initiative aiming to harness AI-driven insights and analytics for disease surveillance, guiding public health decisions, and advancing healthcare outcomes on a global scale. This initiative aggregates millions of de-identified case data points, providing epidemiologists and public health authorities with standardized, openly accessible information to monitor disease transmission and population mobility. In doing so, it has established one of the world's most extensive repositories of publicly available, anonymized COVID-19 data to date.

When considering AI adoption, rigorous evaluation should also be undertaken to assess potential implications. Ensuring unbiased algorithms and inclusive implementation is crucial to harness AI's potential for equitable multidisciplinary collaboration. It is crucial to recognize that diverse contexts result in different AI use cases and goals, while also ensuring that accountability and ethical frameworks address data extraction practices. This includes upholding individual rights, data security, and the use of proper consent protocols to safeguard the ethical use of data in AI applications. For instance, models have so far not employed protocols for handling data scarcity in low-resource regions or subjects, or AI's failures and potential impact on marginalized groups.

It's crucial to recognize that large language models cannot be regarded themselves as generating “correct answers”. The inherent nature of these models is generative, even when capable of producing imaginative content. Their effectiveness is heavily reliant on the quality and accuracy of the data used to train them. If the training data does not faithfully represent reality, the model's outputs may not be genuinely useful. Therefore, we must exercise caution and not solely rely on these tools for achieving a deep understanding of complex issues. Instead, we should use them strategically to address specific challenges. This discussion particularly underscores the responsibility held by those operating in the Global South. Large language models like ChatGPT are predominantly developed in the Global North and are trained on data from platforms that often embed cultural, gender, and race assumptions rooted in the Global North. These implicit biases can extend to structural biases, influencing the model's outputs and potentially perpetuating inequities.

## Perspectives and way forwards

To address these challenges democratized and inclusive approaches are the way forward. Task-specific language models must be co-created with communities, involving them in shaping the dataset itself to ensure it genuinely reflects their culture and context. By democratizing access to AI and ensuring it aligns with diverse needs and perspectives, we can harness its potential to improve operational competence and expand access to essential healthcare services. Using AI to streamline data gathering and analysis could optimize efficiency and frequency, requiring less human effort. This might enhance data generation due to more regular use. In public health, confronting the challenge of producing higher-quality data remains crucial. Whether employing advanced tools or traditional epidemiological methods, this reality persists. Nonetheless, the use of AI but also its shortcomings might prompt a re-evaluation of generating robust public health data globally and across communities. Perhaps, the existence of these tools could inspire innovative approaches to address this imperative.

While the potential applications of AI are undoubtedly intriguing, it is imperative to recognize that AI is not a magic bullet capable of solving every problem effortlessly. Instead, its implementation introduces a set of new challenges, including the critical requirement for extensive and high-quality data, the establishment of robust governance frameworks, and the ongoing need for rigorous evaluation. Successful integration of AI necessitates a holistic approach that goes beyond the allure of its capabilities, emphasizing the importance of addressing these challenges systematically. Key takeaways from this understanding include the need for a realistic perspective on AI's capabilities, the recognition of the essential role of data quality and governance, and an ongoing commitment to continuous evaluation for effective and responsible AI deployment.

In this regard, intentionality is the cornerstone of responsible AI design. Establishing responsible data policies or governance will be a complex endeavour that must align with the unique needs and values of respective communities in different part of the globe. Through principled engagement with policymakers and a commitment to ethically sound AI solutions, this technology has potential to elevate healthcare outcomes, mitigate disparities, and serve as a driving force behind achieving equitable improvements in public health disease surveillance on a worldwide scale.

## Data Availability

Not applicable.

## Abbreviations

WHO: World Health Organization
AI: Artificial Intelligence
AI4PEP: Global South AI for Pandemic & Epidemic Preparedness & Response Network
MSF: Médecins Sans Frontières
AST: Antimicrobial Susceptibility Test
EUCAST: European Committee on Antimicrobial Susceptibility Testing
CLSI: Clinical & Laboratory Standards Institute
